# Post-genomic analyses of fungal lignocellulosic biomass degradation reveal the unexpected potential of the plant pathogen *Ustilago maydis*

**DOI:** 10.1186/1471-2164-13-57

**Published:** 2012-02-02

**Authors:** Marie Couturier, David Navarro, Caroline Olivé, Didier Chevret, Mireille Haon, Anne Favel, Laurence Lesage-Meessen, Bernard Henrissat, Pedro M Coutinho, Jean-Guy Berrin

**Affiliations:** 1 INRA, UMR1163 BCF, 13288 Marseille, France; 2 INRA, UMR1163 CIRM-CF, 13288 Marseille, France; 3 INRA, UMR1319 Micalis, PAPPSO, 78352 Jouy-en-Josas, France; 4 Aix-Marseille Universités, 13288 Marseille, France; 5 CNRS, UMR6098 AFMB, 13288 Marseille, France

**Keywords:** Filamentous fungi, genomes, lignocellulose, enzymatic hydrolysis, cellulases, oxido-reductases, glycosyl hydrolases, *Ustilago maydis*, mass spectrometry

## Abstract

**Background:**

Filamentous fungi are potent biomass degraders due to their ability to thrive in ligno(hemi)cellulose-rich environments. During the last decade, fungal genome sequencing initiatives have yielded abundant information on the genes that are putatively involved in lignocellulose degradation. At present, additional experimental studies are essential to provide insights into the fungal secreted enzymatic pools involved in lignocellulose degradation.

**Results:**

In this study, we performed a wide analysis of 20 filamentous fungi for which genomic data are available to investigate their biomass-hydrolysis potential. A comparison of fungal genomes and secretomes using enzyme activity profiling revealed discrepancies in carbohydrate active enzymes (CAZymes) sets dedicated to plant cell wall. Investigation of the contribution made by each secretome to the saccharification of wheat straw demonstrated that most of them individually supplemented the industrial *Trichoderma reesei *CL847 enzymatic cocktail. Unexpectedly, the most striking effect was obtained with the phytopathogen *Ustilago maydis *that improved the release of total sugars by 57% and of glucose by 22%. Proteomic analyses of the best-performing secretomes indicated a specific enzymatic mechanism of *U. maydis *that is likely to involve oxido-reductases and hemicellulases.

**Conclusion:**

This study provides insight into the lignocellulose-degradation mechanisms by filamentous fungi and allows for the identification of a number of enzymes that are potentially useful to further improve the industrial lignocellulose bioconversion process.

## Background

Lignocellulosic biomass conversion to simple sugars is widely studied for subsequent fermentation to bioethanol or industrial chemicals but biotechnological processes are both complex and costly [1,2]. In the biorefinery process, enzymatic hydrolysis (i.e. saccharification) is one of the major bottlenecks due to the recalcitrance of plant cell wall whose main components, cellulose, hemicellulose and lignin form a tight complex with varying proportions depending on plant species [3,4].

Filamentous fungi are among the most potent degraders of lignocellulosic biomass as they produce a high number and a broad variety of enzymes that have different and complementary catalytic activities [5]. The degradation of lignocellulose by filamentous fungi has been studied in a range of basidiomycetes and ascomycetes. Among wood-decaying basidiomycetes, the white-rot fungi *Phanerochaete chrysosporium *[6] is known to secrete a wide range of enzymes such as lignin peroxidases and glycosyl hydrolases (GHs) [7]. Many ascomycetes species have been identified as good candidates for the release of monosaccharides such as *Trichoderma reesei*, which is used extensively in industry due to its capacity to secrete high level of cellulases. *T. reesei *has undergone several rounds of mutation/selection starting from the QM6a strain. As a result, the engineered *T. reesei *industrial strain CL847 is able to secrete more than 50 g of proteins per litre of culture, which permits a wide-range of applications in different fields of white biotechnology. Additional genetic and biochemical studies have deeply improved our knowledge of *T. reesei *enzymes. More recently, the release of the *T. reesei *genome (QM6a strain) has shown that its carbohydrate active enzyme (CAZyme) machinery is globally comparable to other saprophytic fungi [8-10]. However, compared to other filamentous fungi, the *T. reesei *genome is poor in terms of number and diversity of enzymes that are likely to be involved in biomass degradation [8]. The lack of key lignocellulosic enzymes in *T. reesei *opens opportunities to generate more competitive enzyme cocktails.

During the last decade, large efforts have been concentrated on genome sequencing of ascomycetes, and only a few basidiomycetes genomes were made available. The genomes of phytopathogenic fungi such as *Fusarium graminearum*, a wheat pathogen [11], and *Ustilago maydis*, a maize pathogen [12], have been published. Saprotrophic fungi have also been targeted, e.g., the basidiomycete *P. chrysosporium *[6], the ascomycetes *Neurospora crassa*, *Nectria haematococca *[13,14] and several Aspergilli, such as *Aspergillus nidulans*, *Aspergillus fumigatus*, *Aspergillus oryzae *and *Aspergillus niger *[15-18]. Several other *Aspergillus *genomes (*Aspergillus clavatus*, *Aspergillus fischeri*, *Aspergillus flavus *and *Aspergillus terreus*) have now also been sequenced, and the list continues to grow [19,20].

Although *in silico *annotations of fungal genomes provides large amounts of information about the genes that encode putative lignocellulose-degrading enzymes, experimental analyses are necessary to better understand complex mixture of enzymes that are secreted (i.e. the secretome) in response to inducers. In this study, we thus characterised 20 fungal species for which genomic data are available for their ability to secrete CAZymes targeting plant cell wall by means of high-throughput enzymatic assays and proteomic analyses.

## Results and Discussion

### Genomic analysis of the fungal CAZyme sets dedicated to the plant cell wall

**Figure 1:**
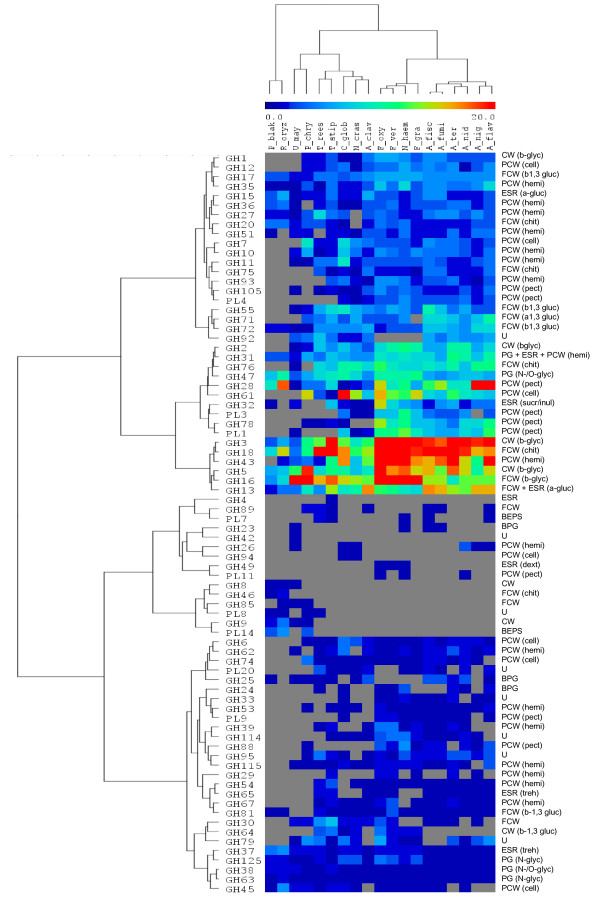
**Comparison of the CAZyme repertoires (Glycoside Hydrolases and Polysaccharide Lyases) identified in the genome of selected fungi using double hierarchical clustering**. Top tree: Fungal genomes analysed (except *M. circ*.). Fungal species names are listed in Table 1. Left tree: Enzyme families represented by their class (GH and PL) and family number according to the carbohydrate-active enzyme database. Right side: Known substrates of CAZy families (most common forms in brackets): BPG, bacterial peptidoglycan; BEPS, bacterial exopolysaccharides; CW, cell wall; ESR, energy storage and recovery; FCW, fungal cell wall; PCW, plant cell wall; PG, protein glycosylation; U, undetermined; a-gluc, α-glucans (including starch/glycogen); b-glyc, β-glycans; b-1,3-gluc, β-1,3-glucan; cell, cellulose; chit, chitin/chitosan; dext, dextran; hemi, hemicelluloses; inul, inulin; N-glyc, N-glycans; N-/O-glyc, N-/O-glycans; pect, pectin; sucr, sucrose; and tre, trehalose. Abundance of the different enzymes within a family is represented by a colour scale from 0 (grey) to ≥ 20 occurrences (red) per species. The figure was edited using the Multiexperiment Viewer software [49].

To determine the sugar-cleaving capabilities of the fungi selected (Table 1), we compared their CAZyme repertoires (GH and polysaccharide lyases, PL) (Figure 1). Clustering of the CAZyme repertoires and the selected fungi resulted in the formation of clusters of fungal species that corresponded with their fungal phyla. As expected, the Aspergilli and Fusaria species have relatively high numbers of GH-encoding genes. In contrast, the *U. maydis *and the zygomycetes *Phycomyces blakesleeanus *and *Rhizopus oryzae *genomes had relatively few genes that encoded for GH. These results indicate different strategies to degrade plant cell wall. The only exception was *A. clavatus *which was grouped with *N. crassa *and *Chaetomium globosum*, both members of the Sordariales order. Interestingly, these three species all share a common feature, as they are known to act on recalcitrant plant material (i.e. are coprophilic).

**Table 1:** Description of strains used in this study.

	Species anamorph (teleomorph*)	Phylum	Family	Original number	Strain ref number	CIRM number
*T.rees*.	*Trichoderma reesei (Hypocrea jecorina)*	*Ascomycota*	*Hypocreaceae*	QM6a	CBS 383.78	BRFM 1104
*P.blak*.	*Phycomyces blakesleeanus*	*Zygomycota*	*Phycomycetaceae*	NRRL 1555	FGSC 10004	BRFM 1098
*R.oryz*.	*Rhizopus oryzae (R. arrhizus)*	*Zygomycota*	*Mucoraceae*	RA 99-880	FGSC 9543	BRFM 1095
*M.circ*.	*Mucor circinelloides *f *lusitanicus*	*Zygomycota*	*Mucoraceae*	NRRL 3631	CBS 277.49	BRFM 1099
*U. may*.	*Ustilago maydis*	*Basidiomycota*	*Ustilaginaceae*	UM521	FGSC 9021	BRFM 1093
*P.chry*.	*Phanerochaete chrysosporium*	*Basidiomycota*	*Phanerochaetaceae*	RP 78	FGSC 9002	BRFM 1090
*T. stip*.	*Penicillium emmonsii (Talaromyces stipitatus)*	*Ascomycota*	*Trichocomaceae*	NRRL 1006	CBS 375.48	BRFM 1102
*A.fisc*.	*Aspergillus fischeri (Neosartorya fischeri)*	*Ascomycota*	*Trichocomaceae*	NRRL 181	CBS 544.65	BRFM 1101
*A.nig*.	*Aspergillus niger*	*Ascomycota*	*Trichocomaceae*	ATCC 1015	FGSC A1144	BRFM 1087
*A.ter*.	*Aspergillus terreus*	*Ascomycota*	*Trichocomaceae*	NIH 2624	FGSC A1156	BRFM 1088
*A.flav*.	*Aspergillus flavus*	*Ascomycota*	*Trichocomaceae*	NRRL 3357	FGSC A1120	BRFM 1086
*A.clav*.	*Aspergillus clavatus*	*Ascomycota*	*Trichocomaceae*	NRRL 1	CBS 513.65	BRFM 1100
*A.fumi*.	*Aspergillus fumigatus*	*Ascomycota*	*Trichocomaceae*	AF 293	FGSC A1100	BRFM 1085
*A.nid*.	*Aspergillus nidulans (Emericella nidulans)*	*Ascomycota*	*Trichocomaceae*	M 139	FGSC A4	BRFM 1084
*F.oxy*.	*Fusarium oxysporum *f *lycopersici*	*Ascomycota*	*Nectriaceae*	NRRL 34936	FGSC 9935	BRFM 1097
*N.haem*.	*Fusarium solani (Nectria haematococca)*	*Ascomycota*	*Nectriaceae*	77-13-4	FGSC 9596	BRFM 1096
*F.gra*.	*Fusarium graminearum (Gibberella zeae)*	*Ascomycota*	*Nectriaceae*	NRRL 31084	FGSC 9075	BRFM 1094
*F. ver*.	*Fusarium verticillioides (Gibberella moniliformis)*	*Ascomycota*	*Nectriaceae*	A-00149	FGSC 7600	BRFM 1089
*C.glob*.	*Chaetomium globosum*	*Ascomycota*	*Chaetomiaceae*	NRRL1870	CBS 148.51	BRFM 1103
*N.cras*.	*Neurospora crassa*	*Ascomycota*	*Sordariaceae*	OR74A	FGSC 9013	BRFM 1092

*when available, **industrial enzymatic cocktail *T. reesei *CL847

### Saccharification efficiencies of fungal secretomes

Hydrolysis of plant biomass requires the production of many different enzymes, which is regulated by the type and complexity of the plant material used as an inducer for the fungal cultures. For example, it has been shown that *Aspergillus *secretomes are strongly tied to the culture conditions and the nutrient sources available [21]. In this study, maize bran was selected as an inducer because (i) it is a natural substrate, (ii) it has been described as being fairly complex and recalcitrant with arabinose and ferulic acid substitutions [22] and (iii) it is a powerful inducer for the expression of a broad range of genes that encode for CAZymes targeting the plant cell wall, e.g., endo-xylanase, endo-mannanase, arabinofuranosidase and carbohydrate esterases [22-24]. All the fungi tested were able to grow on maize bran with a satisfactory yield of secreted protein and were harvested at a single time point, i.e. 7 days of growth (see materials and methods).

Each secretome was diafiltered and concentrated and then tested for its ability to release sugars from micronised wheat straw (WS), taking advantage of the recently developed automated saccharification method [25]. The release of reducing sugars and individual sugar monomers was quantified at the initial rate of hydrolysis (4 h) and at the saccharification plateau (24 h), the *T. reesei *CL847 enzymatic cocktail being used as reference (Table 2).

**Table 2:** Contribution of fungal secretomes to the saccharification of wheat straw.

		*T. rees. CL847*	*T. rees. QM6a*	***P. blak***.	***R. oryz***.	***M. circ***.	***P. chry***.	***U. may***.	***T. stip***.	***A. fisc***.	***A. nig***.	***A. ter***.	***A. flav***.	***A. clav***.	***A. fumi***.	***A. nid***.	***F. oxy***.	***N. haem***.	***F. gram***.	***F. ver***.	***C. glob***.	***N. cras***.
**Initial step (4 h)**																						

+ CL847	**DNS (glucose equivalent)**	0.082	0.106	0.084	0.074	0.083	0.083	0.084	0.112	0.097	0.095	0.086	0.087	0.106	0.112	0.146	0.078	0.078	0.077	0.087	0.090	0.093
	**glucose**	0.071	0.051	0.033	0.036	0.049	0.038	0.037	0.059	0.060	0.091	0.041	0.074	0.051	0.070	0.080	0.037	0.050	0.043	0.043	0.051	0.044

**Plateau (24 h)**																						

- CL847	**DNS (glucose equivalent)**	0.180	0.125	0.086	0.107	0.081	0.063	0.241	0.213	0.136	0.084	0.076	0.060	0.100	0.127	0.187	0.086	0.028	0.109	0.084	0.058	0.115
	**glucose**	0.094	0.033	0.021	0.021	0.018	0.020	0.097	0.032	0.032	0.034	0.017	0.016	0.021	0.035	0.085	0.025	0.020	0.036	0.022	0.026	0.040
	**xylose**	0.042	0.034	0.035	0.030	0.012	0.000	0.034	0.046	0.042	0.023	0.019	0.017	0.026	0.038	0.023	0.020	0.014	0.028	0.000	0.008	0.018

+ CL847	**DNS (glucose equivalent)**	0.181	0.216	0.208	0.209	0.211	0.202	0.285	0.222	0.246	0.222	0.208	0.213	0.227	0.248	0.287	0.213	0.246	0.222	0.208	0.180	0.216
	**glucose**	0.094	0.088	0.091	0.083	0.083	0.078	0.115	0.103	0.105	0.095	0.085	0.081	0.089	0.100	0.119	0.093	0.094	0.092	0.085	0.079	0.089
	**xylose**	0.043	0.041	0.053	0.035	0.034	0.031	0.055	0.048	0.053	0.041	0.034	0.034	0.038	0.047	0.052	0.036	0.045	0.040	0.030	0.032	0.039

Top table, initial step (4 h); Bottom table, plateau (24 h). Total solubilised sugars, glucose and xylose were expressed as glucose equivalent in mg of sugars released per mg of dry matter. Values at 4 h and 24 h are means of sextuplicate and triplicate measures, respectively. Standard errors of the mean were < 5%.

To evaluate the contribution of each secretome to the initial rate of the reaction compared to the *T. reesei *CL847 enzyme cocktail alone, supplementation experiments were performed using the same amount of total enzyme loading (Table 2). As a result, seven secretomes increased significantly the initial rate of the release of reducing sugars. Among them, four secretomes (*A. niger*, *A. flavus*, *A. fumigatus *and *A. nidulans*) were able to increase the glucose rate compared to the *T. reesei *CL847 enzyme cocktail alone. The most striking effects were obtained with *A. nidulans *and *A. niger*, which increased the glucose rate by 1.6- and 1.9-fold, respectively, compared to *T. reesei *CL847.

At the saccharification plateau, each secretome was tested alone and in combination with *T. reesei *CL847 enzyme cocktail. As expected, the improved *T. reesei *CL847 enzyme cocktail released more reducing sugars from WS than the secretome of the *T. reesei *QM6a strain. The glucose and xylose yields were 2.8- and 1.3-fold higher with the *T. reesei *CL847 cocktail than those obtained with the QM6a secretome. The most efficient secretomes, when used alone on micronised WS, were *A. nidulans, Talaromyces stipitatus *and *U. maydis *secretomes, which yielded higher amounts of reducing sugars compared to *T. reesei *CL847 (Table 2). This observation is in agreement with the quantification of the monomers that showed differences in sugar release, i.e., *A. nidulans *and *U. maydis *yielded primarily glucose, whereas *T. stipitatus *yielded mainly xylose. The supplementation of *T. reesei *CL847 with most of the fungal secretomes resulted in a significant increase in the release of soluble sugars, up to a 1.6-fold increase when compared to *T. reesei *alone. A closer look at the monomers indicated that five of the secretomes (*U. maydis*, *T. stipitatus*, *A. fischeri*, *A. fumigatus *and *A. nidulans*) displayed a significant positive effect on both the release of glucose and xylose, while *P. blakesleeanus *only increased the release of xylose. The strongest effect on the release of glucose in the supplementation experiments was obtained when *U. maydis *and *A. nidulans *secretomes were used; they improved the *T. reesei *CL847 enzymatic cocktail by more than 20% (Table 2).

### Enzymatic characterisation of the fungal secretomes using activity profiling

To assess the sugar-cleaving capabilities of all of the fungal secretomes, we quantified their main glycoside-hydrolase activities using a microplate assay that contained para-nitrophenyl (*p*NP)-sugars and complex polysaccharides as substrates (See additional file 1, Table S1). Cellulose degradation was estimated by the quantification of endo-glucanase (carboxy-methyl cellulose, CMC), Avicelase (Avicel, AVI), FPase (Filter paper, FP), cellobiohydrolase (*p*NP-β-D-cellobioside, pCel and *p*NP-β-D-lactobioside, pLac) and β-glucosidase (*p*NP-β-D-glucopyranoside, pGlc) activities. Hemicellulose degradation was estimated by quantifying the xylanases and mannanases using structurally different xylans and mannans as substrates. The main exo-acting glycosidases activities were estimated by the quantification of *p*NP-α-L-arabinofuranoside (pAra), *p*NP-α-D-galactopyranoside (pGal), *p*NP-β-D-xylopyranoside (pXyl) and *p*NP-β-D-mannopyranoside (pMan). Pectic degradation was assessed using arabinogalactan (AGA) and arabinan (ARB) and the overall esterase activity was assessed using *p*NP-acetate (pAc).

Comparison of the *T. reesei *CL847 industrial cocktail with the original *T. reesei *QM6a strain used for genome sequencing revealed (i) a similar cellobiohydrolase and FPase activity, (ii) a 10-fold increase in the endo-glucanase and β-glucosidase activities and (iii) a significant decrease in the xylanase activity (See additional file 1, Table S1). The *A. niger *secretome was, by far, the richest in terms of diversity of enzyme activities measured, except that a low activity towards AVI (4 mU/mg) was observed. AVI was better hydrolysed using the *P. blakesleeanus, P. chrysosporium, F. graminearum *and *N. crassa *secretomes. An overall comparison with the hydrolysis of CMC and FP did not show an obvious trend. For example, the *A. nidulans *secretome displayed an activity towards FP that was comparable with *T. reesei *CL847 and QM6a but was unable to hydrolyse CMC and AVI (See additional file 1, Table S1). With regard to hemicellulose degradation, most of the secretomes were able to significantly hydrolyse all of the tested xylans. Soluble wheat arabinoxylan (SAX) was better hydrolysed than birchwood xylan (BRX) and insoluble wheat arabinoxylan (IAX). The only exception was *A. terreus*, which preferred birchwood xylan. *R. oryzae *appeared to be unable to breakdown significant amount of xylan, as it was recently shown using a range of polysaccharides as carbon sources [26]. Analysis of the correlation between specific activities data and the enzyme composition of secretomes is relatively complicated due to the presence of multi-functional enzymes and synergistic interactions between enzymes [4,27].

**Figure 2:**
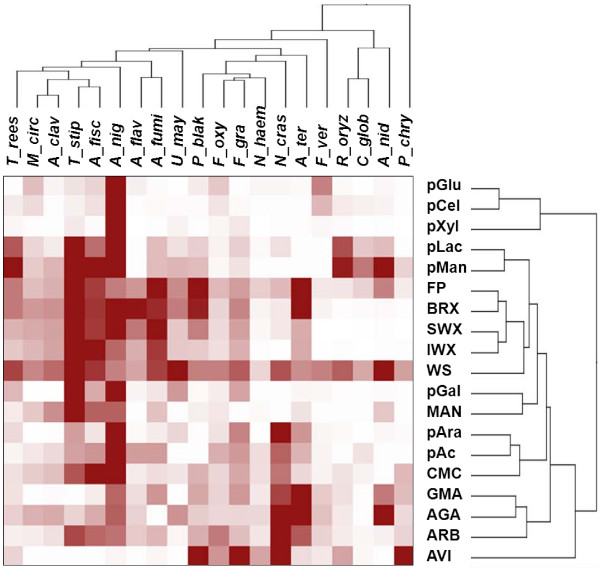
**Double clustering of the main carbohydrate-cleaving activities from representative fungi**. Enzymatic activities of fungal secretomes (except *T. reesei *CL847) on a range of polysaccharides were used to build distance trees. The degree of activity of secretomes on the respective substrate is represented by a colour scale with different strengths of red. Top tree: The fungi names are listed in suppl. Table 1. Right tree: Enzymatic activities of fungal secretomes were determined on pGlc, pCel, pXyl, pLac, pMan, pAra, pGal, pAc, FP, BRX, SAX, IAX, MAN, CMC, AVI, GMA, AGA, ARB and WS as described in material and methods. The figure was edited using the Multiexperiment Viewer software [49].

In order to navigate in the data set and to evaluate the similarities and differences among its components, we performed a double hierarchical clustering (except for the *T. reesei *CL847 enzyme cocktail) with the same bioinformatics tools used to analyse the genomes (Figure 1). Analysis of the full data set identified clusters of strains that showed similar hydrolytic profiles and substrates clusters (Figure 2). *A. niger *was grouped in the cluster that displayed the highest level of activity towards plant cell wall polysaccharides along with four other *Aspergilli *(*A. clavatus*, *A. fischeri*, *A. flavus *and *A. fumigatus*), *T. reesei *QM6a, *Mucor circinelloides *and *T. stipitatus*. However, *A. terreus *and *A. nidulans *did not cluster with the other *Aspergilli. A. nidulans *shares traits in common with *C. globosum *and *R. oryzae*, i.e., a very low activity towards xylans. It is interesting to note that three out of the four *Fusaria *were clustered into the same group and that *P. chrysosporium *did not cluster with any of the other species. Based on data from the literature, wood material or AVI might have been more suitable inducers for *P. chrysosporium *than maize bran and allowed the secretion of a more complete array of plant cell wall degrading enzymes [7,28,29]. Both basidiomycetes *P. chrysosporium *and *U. maydis *activity profiles were rather atypical as they were not correlated together, neither with the other fungi (Figure 2).

When we clustered the substrates on the basis of the activity profiling data, we found that the three xylans tested (BRX, SWX and IWX) clustered together. The correlations observed between the activity on pGlc and pCel can be explained by the fact that both are substrates of β-glucosidases. Although the activity towards cellulosic substrates (CMC, AVI and FP) was not correlated, FP activity was associated with both xylan and WS hydrolysis. These data suggest that FP activity is a better marker than Avicelase activity to estimate the cellulose hydrolysis by fungal secretomes. *P. blakesleeanus*, *T. stipitatus*, *A. fischeri*, *A. terreus*, and *A. fumigatus *secretomes, which all displayed higher FP activities than *T. reesei *CL847 (See additional file 1, Table S1), did not release more glucose from WS (Table 2). Thus, FP activity is not the only factor governing the cellulose hydrolysis efficiency of fungal secretomes. Indeed, it was recently reported that proteins devoid of measurable activity (e.g. GH61) can significantly improve the activity of PCW-degrading enzymes [30,31].

### Proteomic analyses of the best performing fungal secretomes

Recently, there has been growing interest for the detailed analysis of fungal secretomes [28,29,32-34]. Liquid chromatography-tandem mass spectrometry (LC-MS/MS) proteomic analysis is a tool of great interest to attempt to make available the relative abundances of their different protein components. Based on saccharification data, the best-performing secretomes, i.e. *A. niger, A. nidulans and U. maydis*, were selected to be analysed in depth using 1D LC-MS/MS along with the *T. reesei *CL847 enzymatic cocktail. *A. niger *was further investigated due to its capacity to potentiate *T. reesei *CL847 enzymatic cocktail activity at the initial step of hydrolysis whereas *A. nidulans *and *U. maydis *were selected for their boosting effect observed at the saccharification plateau.

Proteomic analysis of the *T. reesei *CL847 enzymatic cocktail allowed the identification of 32 proteins of which 27 were CAZymes targetting plant cell wall, e.g., CBHI (GH7) and CBHII (GH6)/EGI (GH7), EGII (GH5), EGIII (GH12), EGIV (GH61) and EGVI (GH74)/BGLI (GH3)/XYNII (GH11) and XYNIII (GH10)/BXLI (GH3)/ABFI (GH54), ABFII (GH62) and ABFIII (GH54) (See additional file 2, Table S2). These results are in agreement with the proteomic analysis of *T. reesei *CL847 secretome using 2D gel that allowed the identification of 22 proteins [35]. It is interesting to note that our proteomic analysis of *T. reesei *CL847 enzymatic cocktail also showed that the proteins displaying a family 1 carbohydrate binding module (CBM) were among the most abundant protein of the enzymatic cocktail (See additional file 2, Table S2).

**Figure 3:**
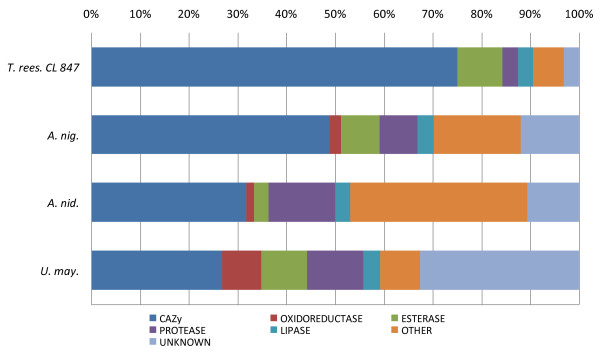
**Enzyme class distribution of proteins identified by LC-MS/MS in secretomes from *T. reesei *CL847, *A. niger*, *A. nidulans *and *U. maydis***. CAZymes correspond to GH and PL families. The bar size indicates the percentage of enzymes of the total of secreted proteins.

Concerning the fungal species selected for proteomic analysis, a total of 242, 66 and 86 proteins were detected from the concentrated secretomes of *A. niger*, *A. nidulans *and *U. maydis*, respectively, by mass-matching against corresponding protein databases. The *A. niger *secretome contained an impressive array of 118 CAZymes out of the total 242 proteins (Figure 3). The number of exo-acting glycosidases identified in the secretome of *A. niger *when cultured in the presence of maize bran was disproportionally higher than the number of endo-acting CAZymes (See additional file 2, Table S2). This observation corresponds with a previous proteomic analysis on the *A. niger *secretome under different cultured environments [34]. The presence of a broad array of exo-acting glycosidases might be responsible for the significant increase in the initial rate of saccharification when the *A. niger *secretome was added to the *T. reesei *CL847 enzyme cocktail. Surprisingly, this positive effect was not observed at the saccharification plateau, indicating that the large diversity and high number of CAZymes do not act in concert with the *T. reesei *enzymes during the saccharification of WS. Compared to *A. niger*, the *A. nidulans *secretome displayed a lower enzymatic diversity towards plant cell walls. This striking difference could be due to the regulatory mechanisms of enzyme induction, which are controlled by the xylose and arabinose transcriptional activators *XlnR *and *AraR *that differ between *A. nidulans *and *A. niger *[19,36,37]. Of the 19 GHs identified in the *A. nidulans *secretome, we identified seven hemicellulases from the GH10, GH11, GH39, GH43, GH62 and GH93 families and five β-1,3 glucanases from the GH17, GH55 and GH81 families (See additional file 2, Table S2). The absence of activity on CMC and AVI correlated with the fact that no endo β-1,4 glucanase or cellobiohydrolase was identified by proteomics. However, the high activities of the *A. nidulans *secretome on FP and WS are rather intriguing (See Figure 2 and additional file 1, Table S1). This observation might suggest the presence of novel specificities among the plant cell wall degrading enzymes.

The analysis of *U. maydis *secretome revealed a total of 86 proteins (see additional file 2, Table S2). They represent more than half of the theoretically secreted proteins as *U. maydis *genome is supposed contains 168 genes that encode for secreted proteins [38]. The *U. maydis *genome contains one of the smallest sets of genes that encode for CAZymes with 95 GH compared to the genome of necrotrophic plant pathogenic fungi (Figure 1) [12,38]. Of the 86 proteins identified in the *U. maydis *secretome, we identified 23 CAZymes that target the plant cell wall. Of the most abundant proteins, we identified one GH10 xylanase and two GH51 and GH62 arabinofuranosidases. Interestingly, *T. reesei *genome has the smallest set of arabinofuranosidases of all of the plant cell wall-degrading ascomycetes. Thus, *U. maydis *hemicellulases may act in synergy with *T. reesei *enzymes to efficiently depolymerise wheat arabinoxylan, thereby facilitating cellulose access for the *T. reesei *cellulases. Another striking difference with the other fungal proteomes analysed, is the significant fraction of the *U. maydis *secretome (i.e. 8% of the total protein) of putative oxido-reductases that are potentially involved in the depolymerisation of lignocellulose. Two of the most abundant proteins identified corresponded to putative glucose-methanol-choline (GMC) oxidoreductases (Q4P967 and Q4P769), which are FAD flavoproteins with diverse catalytic activities and two others proteins are known as glyoxal oxidases (Q7Z866 and Q7Z867). Interestingly, *U. maydis *glyoxal oxidases have been experimentally shown to be essential for successful maize infection [39]. Although the role of fungal oxido-reductases in biomass deconstruction remains unclear, their presence suggest that, via the formation of highly reactive oxidants, they could participate in the depolymerisation of lignocellulose, as previously suggested for the wood-decaying fungus *Postia placenta *[40]. Indeed, glyoxal and glucose oxidases are known to generate H_2_O_2 _that is supposed to play a role in lesion formation and lesion expansion of plant cell wall in the infection mechanism of fungal pathogens [41]. As *T. reesei *is devoid of oxido-reductases, some of the putative *U. maydis *oxidases identified in its secretome are likely responsible for the observed increase in saccharification efficiency. Recent data from literature on the synergy between GH61 and oxido-reductases [42] might also explain the efficiency of *U. maydis *secretome in combination with *T. reesei *that contains a GH61 enzyme (EGIV). Recently, there has been growing interest in the potential of plant-pathogenic fungi to optimise the enzymatic treatment processes for the optimal hydrolysis of lignocellulosic biomass [43]. A recent large-scale screening using 156 ascomycetes revealed that the plant pathogens were more active than the non-pathogens on several lignocellulosic substrates [44]. *U. maydis *is a well characterised basidiomycete fungus that parasitises maize. It belongs to the smut fungi, which share a biotrophic mode of growth, i.e., they grow on the host plant without killing it. To our knowledge, the maize pathogenic basidiomycete *U. maydis *has never been tested for its hydrolysis potential of lignocellulosic biomass. The use of maize bran as an inducer is likely a key factor that contributed to the induction of the specific enzymes that target the plant cell walls of monocots (maize and wheat). The enzymes identified by the proteomic analysis of the secretome of *U. maydis *may facilitate access to cellulose and hemicellulose for the *T. reesei *CL847 enzymes, thereby explaining the increase in the glucose and xylose yields.

## Conclusions

The discovery of fungal enzymes that display novel specificities is essential to efficiently breakdown lignocellulosic biomass for the production of biofuels and other high-value products. This post-genomic approach revealed the unexpected potential of *U. maydis*, which contained unique enzymatic equipment that significantly supplemented the *T. reesei *enzymatic cocktail. The putative *U. maydis *oxido-reductases could play a crucial role in improving the hydrolysis of plant cell walls. Future studies of the synergies between hydrolytic enzymes and oxido-reductases are necessary to give additional insights into the filamentous fungi enzymatic machineries.

## Methods

### Annotation of Carbohydrate Active enZymes (CAZymes)

To compare the sugar-cleaving capabilities of the fungi selected, we have listed the number of representatives of each of the glycoside hydrolases (GH) and polysaccharide lyases (PL) families (defined in the carbohydrate active enzyme database (CAZy database) http://www.cazy.org; [9] and then performed a double clustering based on Bray-Curtis distances (i) between organisms according to their family distribution and (ii) between families according on their distribution pattern in the different genomes. Distances were computed using the multivariate analysis programme GINKGO [45]. The distance matrixes were computed using the Species Profiles Distance model and analysed by a hierarchical agglomerative clustering method.

### Fungal strains and enzymatic preparations

The fungal strains used in this study were obtained from the CBS-KNAW (Centraalbureau voor Schimmelcultures, Utrecht, The Netherlands) and the FGSC (Fungal Genetics Stock Center, Kansas City, USA) fungal collections (Table 1). They were streaked and verified by ITS (Internal Transcribed Spacer) sequencing and maintained in the culture collection entitled "Centre International de Ressources Microbiennes", which is dedicated to filamentous fungi of biotechnological interest (CIRM-CF; http://www.inra.fr/crb-cirm/), at the National Institute of Agricultural Research (INRA), Marseille, France. The strains were maintained on malt agar slants, using as MA2 (malt extract at 2% w/v) medium for basidiomycetes and MYA2 (malt extract at 2% w/v and yeast extract at 0.1% w/v) medium for ascomycetes and zygomycetes. The *T. reesei *CL847 secretome (E508 enzymatic cocktail) obtained from IFPEN (Rueil-Malmaison, France) was used as a reference enzymatic cocktail [24,46].

### Culture conditions and secretome preparation

Based on previous studies [22,23], the fungal cultures were grown in a liquid medium containing 15 g.l^-1 ^(based on the dry matter) of the autoclaved maize bran fraction (provided by ARD, Pomacle, France) as a carbon source, 2.5 g.l^-1 ^of maltose as a starter, 1.842 g.l^-1 ^of diammonium tartrate as a nitrogen source, 0.5 g.l^-1 ^yeast extract, 0.2 g.l^-1 ^KH_2_PO_4_, 0.0132 g.l^-1 ^CaCl_2_.2H_2_O and 0.5 g.l^-1 ^MgSO_4_.7H_2_O. The sugar content (w/w) of the autoclaved maize bran fraction is 16.10% arabinose, 28.73% xylose, 0.17% mannose, 5.65% galactose and 22.06% glucose. Miniaturized fungal cultures were carried out in 16-well baffled plates as described in [47]. The cultures were inoculated with 2 × 10^5 ^spores.ml^-1 ^for sporulating fungi, or with mycelial fragments generated using a Fastprep^®^-24 (MP Biomedicals) set to 5 m.s^-1 ^for 40 s, for non-sporulating fungi prior to incubation in 16-well baffled plates at 30°C with orbital shaking at 140 rpm (Infors HT, Switzerland).

All of the cultures were stopped at 7 days after inoculation. The cultures were stopped at 7 days after inoculation since (i) it is the mid-term growth for most of fungi (basidiomycetes and ascomycetes) in our conditions (16-well baffled plates) and (ii) extended growth led to evaporation of the culture medium. The culture broth (secretome) was harvested (total volume of 20 to 30 ml), filtered (using 0.2 μm polyethersulfone membrane, Vivaspin, Sartorius), diafiltered and concentrated (Vivaspin with a 10 kDa cut-off polyethersulfone membrane, Sartorius) in 50 mM acetate solution buffer, pH 5 to a final volume of 3 ml and then stored in appropriate vials (1.2-ml tubes with septa in cluster plate, ABgene, Thermo scientific, USA) at -20°C until use.

The total amount of protein was assessed using Bradford assays (Bio-Rad Protein Assay Dye Reagent Concentrate, Ivry, France) with a BSA standard that ranged from 0.2 to 1 mg.ml^-1^.

### Saccharification assays on micronised lignocellulosic substrates

The concentrated secretomes were tested for their ability to hydrolyse micronised WS (*Triticum aestivum*, Apache, France), which was prepared by G. Ghizzi and X. Rouau as described in [25]. WS particles had an average size of 100 μm. A 1% (w/v) WS suspension was prepared in 50 mM acetate buffer, pH 5, supplemented with 40 μg.ml^-1 ^of tetracycline as an antibiotic and 30 μg.ml^-1 ^of cycloheximide as an antifungal agent. The resulting suspension was dispensed into 96-well plates by the Tecan Genesis Evo 200 robot (Tecan, Lyon, France) and the plates were frozen at -20°C until needed.

The saccharification assay was performed using a high-throughput automated method that has been previously described [25] using a Tecan Genesis Evo 200 robot (Tecan). All of the appropriate blanks and controls reactions were performed as described in [25]. The initial step of wheat-straw hydrolysis was measured after 4 hours using normalized amount of protein (20 μg of *T. reesei *CL847 enzymatic cocktail and 10 μg of each secretome). The control reaction was performed with 30 μg of *T. reesei *CL847 enzymatic cocktail. Saccharification was performed at 37°C with 8 Hz shaking. After 4 hours of incubation, the saccharification reactions were filtered and recovered; the reducing sugars were quantified using the DNS method and the glucose content was measured using a Glucose RTU kit (Biomérieux, Marcy l'Etoile, France) following the manufacturer's instructions. All of the reactions were performed independently at least six times.

To quantify the sugars released at the saccharification plateau (24 h for WS), 15 μl of concentrated secretome (5 to 30 μg total proteins) was added to the substrate plate either alone or in addition to 30 μg of the *T. reesei *CL847 enzymatic cocktail. The released reducing sugars were measured by DNS assay. The saccharification reactions were also analysed by high performance anion exchange chromatography (Dionex, Voisins-le-Bretonneux, France) and a CarboPac PA-1 column (Dionex) to quantify the amounts of glucose and xylose, as described in [24]. All of the reactions were independently performed at least in triplicate.

### Enzyme activity measurements

*p*NP-based chromogenic substrates and complex substrates were used to assay the enzymatic activities of the fungal secretomes. pGlc, pLac, pCel, pXyl, pAra, pGal, pMan and pAc were obtained from Sigma (Sigma-Aldrich, St.Louis, MO). For all *p*NPs except for pAc, a 1 mM *p*NP solutions was prepared in 50 mM sodium acetate buffer pH 5, and distributed into polystyrene 96-well plates (IWAKI, Japan) at 100 μl per well and one column per substrate. *p*NP standards ranging from 0 to 0.2 mM were then added to the plates. The plates were frozen at -20°C until use. The assay was performed by adding appropriate dilutions of 20 μl of the secretome to *p*NP plates that had been previously preincubated at 37°C. The plates were sealed using a PlateLoc plate sealer (Velocity 11, Agilent) to prevent evaporation, and incubated at 37°C with shaking at 1000 rpm (Mixmate, Eppendorf). After 30 minutes, the reaction was stopped by the addition of 130 μl of 1 M Na_2_CO_3 _solution pH 11.5. The amount of *p*NP released was measured at 410 nm and quantified using a *p*NP standard curve ranging from 0 to 20 nmol. As pAc is unstable at room temperature in acetate buffer, pH 5, the stock solution of pAc was prepared at 20 mM in DMSO and diluted to 1 mM in 50 mM sodium phosphate, pH 6.5, just prior to use. We immediately added 15 μl of the secretome, and the kinetics of hydrolysis were followed at 410 nm for one minute. One unit of enzyme was defined as 1 μmol of *p*-nitrophenyl released per mg of enzyme per minute under our experimental conditions.

The complex substrates used in this study were carboxymethyl cellulose (CMC, Sigma), Avicel PH101 (Fluka), birchwood xylan (Sigma), low viscosity wheat arabinoxylan (Megazyme, Wicklow, Ireland), insoluble wheat arabinoxylan (Megazyme), insoluble ivory nut mannan (MAN, Megazyme), carob galactomannan (GMA, Megazyme), larch wood arabinogalactan (Megazyme) and sugar beet arabinan (Megazyme). A 1% w/v suspension/solution was prepared in 50 mM sodium acetate buffer, pH 5, and distributed into 96-well plates at 100 μl per well and one column per substrate. Glucose standards that ranged from 0 to 20 mM were added to the plate. The plates were then frozen at -20°C until use. The assay was performed by adding 20 μl of the secretome (at three appropriate dilutions) to the previously thawed substrate plates. The plates were shaken and incubated at 37°C for one hour using the Tecan Genesis Evo 200 robot plate incubator (Tecan France, Lyon, France), and the reducing sugars were quantified using the automated DNS method, as described in [25]. One unit of enzyme was defined as 1 μmol of glucose equivalent released per mg of enzyme per minute under the assay conditions used here.

The global cellulase activity was determined on filter paper (Whatmann n°1) using 6 mm diameter discs using a protocol adapted from [48]. Vials containing one filter paper disc in 100 μl of 50 mM sodium acetate buffer, pH5, and 50 μl of the secretome were incubated for 2 hours at 50°C. All of the assays were performed in triplicate. After incubation, the reducing sugars were quantified using the automated DNS method [25] with glucose standards that ranged from 0.2 to 20 mM. One unit of enzyme was defined as 1 μmol of glucose equivalent released per mg of enzyme per minute under our experimental conditions.

Data from the activities measured in all the secretomes except *T. reesei *CL847 were ordered and grouped using the same clustering method as described for the genome analysis of CAZymes.

### Identification of proteins by LC-MS/MS analysis

Proteins from the diafiltered supernatants of *A. niger*, *A. nidulans*, *U. maydis *and *T. reesei *CL847 (25 μg) were separated by one-dimensional (1D) electrophoresis (Precast Tris-Glycine 12% SDS-PAGE gels, BioRad) and stained with Coomassie blue (BioRad). Each 1D electrophoresis lane was cut into 24 gel fragments (2 mm in width) and protein identification was performed using the PAPPSO platform facilities (http://pappso.inra.fr). In-gel digestion was carried out with the Progest system (Genomic Solution) according to a standard trypsinolysis protocol. Gel pieces were first washed twice with 10% (v/v) acetic acid followed by a wash with 40% (v/v) ethanol in water and then with acetonitrile. They were then further washed with 25 mM NH_4_CO_3 _and dehydrated in acetonitrile (two alternating cycles). Digestion was performed for 6 hours at 37°C with 125 ng of modified trypsin (Promega) dissolved in 20% (v/v) methanol in 20 mM NH_4_CO_3_. The tryptic peptides were first extracted with 50% (v/v) acetonitrile and 0.5% trifluoroacetic acid in water and then with pure acetonitrile. The two peptide extracts were pooled, dried in a vacuum speed concentrator and suspended in 25 μl of 2% (v/v) acetonitrile and 0.08% (v/v) trifluoroacetic acid in water.

LC-MS/MS analysis was performed on an Ultimate 3000 LC system (Dionex) connected to an LTQ-Orbitrap Discovery mass spectrometer (Thermo Fisher, USA) using a nanoelectrospray ion source. After 4 min, the precolumn was connected to the separating nanocolumn Pepmap C18 (0.075 × 15 cm, 100Å, 3 μm) and the linear gradient was started from 2 to 36% of buffer B (80% acetonitrile and 0.1% formic acid) in buffer A (2% acetonitrile and 0.1% formic acid) at 300 nl.min^-1 ^for 50 min. The doubly and triply charged precursor ions were subjected to MS/MS fragmentation with a 1-min exclusion window and classical peptide fragmentation parameters (Qz = 0.22, activation time = 50 ms, collision energy = 35%).

The raw mass data were first converted to the mzXML format with the ReAdW software (http://tools.proteomecenter.org/software.php). Protein identification was performed by querying the MS/MS data against the corresponding protein databases (UniProtKB, 2009.06.08) along with an in-house contaminant database, using the X!Tandem software (X!Tandem Tornado 2008.02.01.3, http://www.thegpm.org) with the following parameters: one missed trypsin cleavage, alkylation of cysteine and conditional oxidation of methionine precursor and fragment ion set to 10 ppm and 0.5 Da, respectively. A refined search was added with similar parameters, except that the semi-tryptic peptides and the possibly N-terminal acetylated proteins were included. All the peptides that matched with an E-value lower than 0.05 were parsed with an in-house programme (http://http:/PAPPSO.inra.fr/bioinformatique.html). Proteins identified by at least two unique peptides and a log (E-value) lower than 1.E-8 were considered to be validated. A list of proteins and peptides with masses and log (E-value) is provided for *T. reesei*, *A. niger*, *A. nidulans *and *U. maydis *proteomes in additional files 3, 4, 5 and 6 (Tables S3, S4, S5, S6), respectively.

## List of abbreviations

AVI: Avicel; AGA: arabinogalactan; ARB: arabinan; BRX: birchwood xylan; CAZy: Carbohydrate Active enZyme database; CAZyme: carbohydrate-active enzyme; CBM: carbohydrate binding module; CMC: carboxy-methyl cellulose; FP: filter paper; GH: glycosyl hydrolase; GMA: galactomannan; IAX: insoluble arabinoxylan; LC-MS/MS: liquid chromatography-tandem mass spectrometry; MAN: ivory nut mannan; pAc: *p*NP-acetate; pAra: *p*NP-α-L-arabinofuranoside; pCel: *p*NP-β-D-cellobioside; pGal: *p*NP-α-D-galactopyranoside; pGlc: *p*NP-β-D-glucopyranoside; PL: polysaccharide lyases; pLac: *p*NP-β-D-lactobioside; pMan: *p*NP-β-D-mannopyranoside; *p*NP: *Para*-nitrophenyl; pXyl: *p*NP-β-D-xylopyranoside; SAX: soluble wheat arabinoxylan; WS: wheat straw.

## Competing interests

The authors declare that they have no competing interests.

## Authors' contributions

MC carried out fungal cultures, enzyme and saccharification assays and helped to draft the manuscript. CO, DN and MH helped to carry out experimental work. DC performed LC-MS/MS analyses and DN participated to the annotation of proteomes. DN, AF, LLM, BH, PMC and JGB participated in the design of the study. PMC and BH performed comparative genomic analysis and annotation of proteome. JGB and PMC conceived the study and coordinated and supervised the analysis. JGB drafted and submitted the manuscript. All authors read and approved the final manuscript.

## Supplementary Material

Additional file 1 **Activity profiling of fungal secretomes**. The file contains a table with enzymatic activities (mU/mg) that were obtained using *p*NP-based substrates and complex substrates as described in materials and methods. Values are means of triplicate independent measures. Click here for file

Additional file 2 **List of proteins confidently identified in the *T. reesei *CL847 enzymatic cocktail and the secretome of *A. niger, A. nidulans *and *U. maydis *using 1D LC-MS/MS analyses**. The file contains an Excel table that includes SDS-Page of the *T. reesei *CL847 enzymatic cocktail and the secretome of *A. niger*, *A. nidulans *and *U. maydis *and a list of the proteins that have been confidently identified in the four samples using 1D LC-MS/MS analyses and annotation of putative function. For CAZymes (GH and PL) and CBM, families are indicated. Click here for file

Additional file 3 **Detailed list of the mass data of peptides identified in the *T. reesei *CL847 enzymatic cocktail using 1D LC-MS/MS analyses**. The file contains an Excel table with data of peptides identified Click here for file

Additional file 4 **Detailed list of the mass data of peptides identified in the *A. niger *secretome using 1D LC-MS/MS analyses**. The file contains an Excel table with data of peptides identified Click here for file

Additional file 5 **Detailed list of the mass data of peptides identified in the *A. nidulans *secretome using 1D LC-MS/MS analyses**. The file contains an Excel table with data of peptides identified Click here for file

Additional file 6 **Detailed list of the mass data of peptides identified in the *U. maydis *secretome using 1D LC-MS/MS analyses**. The file contains an Excel table with data of peptides identified Click here for file
