# Supplementary material for: Post-genomic analyses of fungal lignocellulosic biomass degradation reveal the unexpected potential of the plant pathogen Ustilago maydis
Source: BMC Genomics. 2012 Feb 2;13:57. doi: 10.1186/1471-2164-13-57 (PMC3298532; doi:10.1186/1471-2164-13-57)
Supplement: Additional file 1 — Activity profiling of fungal secretomes. The file contains a table with enzymatic activities (mU/mg) that were obtained using pNP-based substrates and complex substrates as described in materials and methods. Values are means of triplicate independent measures. [file 1471-2164-13-57-S1.DOC]

**Supplementary file 1.**

**Activity profiling of fungal secretomes.** Enzymatic activities (mU/mg) were obtained using *p*NP-based substrates and complex substrates as described in materials and methods. Values are means of triplicate independent measures.

| Substrates | pGlu | pLac | pCel | pXyl | pAra | pGal | pMan | pAc | FP | CMC | AVI | BRX | SAX | IAX | MAN | GMA | ARB | AGA |
| --- | --- | --- | --- | --- | --- | --- | --- | --- | --- | --- | --- | --- | --- | --- | --- | --- | --- | --- |
| Strains |
| *T. rees. CL847* | 215 | 41 | 48 | 7 | 26 | 9 | 0 | 5 | 115 | 585 | 69 | 1627 | 2436 | 1034 | 106 | 311 | 5 | 0 |
| *T. rees. QM6a* | 20 | 48 | 45 | 64 | 232 | 648 | 35 | 48 | 101 | 34 | 16 | 4017 | 5092 | 1319 | 12 | 22 | 31 | 78 |
| *P. blak.* | 148 | 14 | 12 | 0 | 27 | 63 | 9 | 13 | 197 | 85 | 120 | 6673 | 8682 | 1280 | 9 | 64 | 156 | 84 |
| *R. oryz.* | 46 | 50 | 48 | 37 | 34 | 39 | 30 | 0 | 20 | 32 | 34 | 137 | 59 | 0 | 0 | 23 | 38 | 37 |
| *M. circ.* | 481 | 12 | 82 | 28 | 10 | 11 | 7 | 94 | 49 | 57 | 0 | 3035 | 5753 | 1714 | 30 | 0 | 37 | 131 |
| *U. may.* | 7 | 9 | 11 | 98 | 249 | 932 | 10 | 11 | 88 | 30 | 0 | 3936 | 3752 | 1295 | 0 | 0 | 20 | 180 |
| *P. chry.* | 0 | 0 | 0 | 1 | 28 | 0 | 6 | 0 | 15 | 78 | 133 | 136 | 31 | 0 | 0 | 48 | 84 | 63 |
| *T. stip.* | 214 | 74 | 34 | 174 | 586 | 3310 | 48 | 350 | 272 | 190 | 0 | 10816 | 22703 | 8524 | 199 | 0 | 354 | 73 |
| *A. fisc.* | 327 | 42 | 70 | 41 | 166 | 791 | 40 | 405 | 146 | 401 | 0 | 6063 | 13311 | 5735 | 87 | 14 | 292 | 138 |
| *A. nig.* | 2678 | 103 | 787 | 2737 | 2150 | 2398 | 46 | 1358 | 98 | 731 | 4 | 10489 | 20137 | 3544 | 87 | 113 | 271 | 267 |
| *A. ter.* | 42 | 0 | 0 | 31 | 630 | 84 | 0 | 287 | 181 | 44 | 13 | 8246 | 5534 | 2004 | 16 | 264 | 319 | 496 |
| *A. flav.* | 121 | 0 | 9 | 1 | 43 | 109 | 0 | 372 | 79 | 23 | 0 | 7027 | 9170 | 1900 | 0 | 25 | 102 | 76 |
| *A. clav.* | 84 | 6 | 9 | 17 | 16 | 15 | 10 | 143 | 60 | 70 | 0 | 3253 | 6287 | 2159 | 62 | 4 | 61 | 139 |
| *A. fumi.* | 38 | 19 | 14 | 26 | 117 | 240 | 9 | 374 | 197 | 38 | 4 | 6408 | 24349 | 4665 | 34 | 77 | 139 | 0 |
| *A. nid.* | 61 | 19 | 30 | 26 | 49 | 47 | 45 | 0 | 95 | 0 | 0 | 207 | 236 | 62 | 31 | 98 | 29 | 581 |
| *F. oxy.* | 422 | 4 | 59 | 67 | 170 | 177 | 1 | 302 | 53 | 47 | 39 | 1313 | 2562 | 776 | 14 | 32 | 257 | 68 |
| *N. haem.* | 71 | 2 | 6 | 2 | 8 | 0 | 0 | 128 | 38 | 36 | 33 | 377 | 530 | 90 | 4 | 18 | 50 | 67 |
| *F.gra.* | 16 | 14 | 14 | 195 | 742 | 852 | 4 | 383 | 74 | 98 | 104 | 3661 | 6438 | 1878 | 0 | 76 | 125 | 69 |
| *F. ver.* | 931 | 0 | 158 | 61 | 56 | 71 | 0 | 47 | 19 | 15 | 9 | 396 | 43 | 25 | 0 | 39 | 87 | 103 |
| *C. glob.* | 7 | 16 | 34 | 7 | 6 | 29 | 18 | 110 | 34 | 15 | 3 | 163 | 63 | 10 | 0 | 3 | 16 | 0 |
| *N. cras.* | 64 | 9 | 0 | 0 | 1415 | 0 | 0 | 589 | 52 | 154 | 100 | 711 | 1561 | 23 | 0 | 138 | 697 | 599 |
